# Patterns of pathologic lymph nodes in anal cancer: a PET-CT-based analysis with implications for radiotherapy treatment volumes

**DOI:** 10.1186/s12885-021-08187-8

**Published:** 2021-04-22

**Authors:** Anna Frennered, Jonas Scherman, Pamela Buchwald, Anders Johnsson, Hanna Sartor, Sophia Zackrisson, Elin Trägårdh, Martin P. Nilsson

**Affiliations:** 1 Diagnostic Radiology, Department of Translational Medicine, Skåne University Hospital, Lund University, Malmö, Sweden; 2 Radiation Physics, Department of Hematology, Oncology and Radiation Physics, Skåne University Hospital, Lund, Sweden; 3 Department of Surgery, Colorectal Unit, Skåne University Hospital, Malmö, Sweden; 4 Department of Hematology, Oncology and Radiation Physics, Skåne University Hospital, Lund, Sweden; 5 Clinical Physiology and Nuclear Medicine, Skåne University Hospital, Lund University, Malmö, Sweden; 6 Division of Oncology and Pathology, Department of Clinical Sciences, Lund University, Lasarettsgatan 23, Skåne University Hospital, S-221 85 Lund, Sweden

**Keywords:** Anal cancer, Anal carcinoma, PET-CT, Lymph node metastasis, Contouring guidelines

## Abstract

**Background:**

This study investigates the patterns of PET-positive lymph nodes (LNs) in anal cancer. The aim was to provide information that could inform future anal cancer radiotherapy contouring guidelines.

**Methods:**

The baseline [18F]-FDG PET-CTs of 190 consecutive anal cancer patients were retrospectively assessed. LNs with a Deauville score (DS) of ≥3 were defined as PET-positive. Each PET-positive LN was allocated to a LN region and a LN sub-region; they were then mapped on a standard anatomy reference CT. The association between primary tumor localization and PET-positive LNs in different regions were analyzed.

**Results:**

PET-positive LNs (*n* = 412) were identified in 103 of 190 patients (54%). Compared to anal canal tumors with extension into the rectum, anal canal tumors with perianal extension more often had inguinal (*P* < 0.001) and less often perirectal (*P* < 0.001) and internal iliac (*P* < 0.001) PET-positive LNs. Forty-two patients had PET-positive LNs confined to a solitary region, corresponding to first echelon nodes. The most common solitary LN region was inguinal (25 of 42; 60%) followed by perirectal (26%), internal iliac (10%), and external iliac (2%). No PET-positive LNs were identified in the ischiorectal fossa or in the inguinal area located posterolateral to deep vessels. Skip metastases above the bottom of the sacroiliac joint were quite rare. Most external iliac PET-positive LNs were located posterior to the external iliac vein; only one was located in the lateral external iliac sub-region.

**Conclusions:**

The results support some specific modifications to the elective clinical target volume (CTV) in anal cancer. These changes would lead to reduced volumes of normal tissue being irradiated, which could contribute to a reduction in radiation side-effects.

**Supplementary Information:**

The online version contains supplementary material available at 10.1186/s12885-021-08187-8.

## Introduction

Anal cancer is a rare malignancy representing approximately 2–3% of all gastrointestinal cancers, but the incidence is increasing [1–4]. Standard treatment of anal cancer is radiotherapy with concurrent chemotherapy, which leads to a cure in 60–80% of the cases [5–7]. However, late side-effects are common mainly due to the radiotherapy [8]. To further improve the treatment outcomes of anal cancer, optimization of the radiotherapy is important. This includes implementation of new techniques such as intensity-modulated radiotherapy (IMRT), which has been shown to reduce acute and chronic radiation-related toxicities [9]. Another line of development is to refine the target delineation, particularly regarding lymph nodes (LNs) included in the elective clinical target volume (CTV).

Contemporary guidelines recommend baseline tumor staging with both magnetic resonance imaging (MRI) and [18F]-fluorodeoxyglucose positron emission tomography with computed tomography (PET-CT) [10, 11]. PET-CT has a high sensitivity in identifying pathologic LNs because most anal cancers are FDG-avid [10, 12]. By these investigations LN metastases are identified, but they do not provide any information on which elective LN regions should be included in the individual patient. For radiotherapy target delineation in clinical practice, several different guidelines have been published [13–16]. To further improve the delineation guidelines in a more risk-adapted way, better knowledge on the routes of lymphatic spread is needed.

LN recurrences within the irradiated volume are very infrequent following chemoradiotherapy of anal cancer. In the largest patterns of recurrence study to date, Shakir et al. reported that only 7 out of 385 patients had a pelvic LN recurrence [17]. Accordingly, ‘gold standard’ patterns of recurrence studies could be used to investigate local and distant recurrences, but for detailed information on regional LN metastasis other types of studies are needed, e.g. studies using pretherapeutic images.

The aim of our present study was to investigate the patterns of PET-positive LNs in anal cancer. We retrospectively assessed the baseline PET-CTs of 190 consecutive anal cancer patients. We defined LNs with a Deauville score (DS) of ≥3, meaning an uptake exceeding that of the mediastinal blood pool, as PET-positive [18, 19].

## Methods and materials

### Study population and data collection

The study population has previously been described in detail [20]. Briefly, all patients with squamous cell carcinoma of the anal region (anal cancer) treated with radiotherapy at the Skåne University Hospital in Lund, August 1st, 2009 – December 31st, 2017 were selected from an institutional database (*n* = 203). Following exclusion of patients in whom a baseline PET-CT had not been performed at the Skåne University Hospital, 190 patients remained. The baseline PET-CTs for these 190 patients were re-evaluated by a senior radiologist (AF). Complicated cases were reviewed by a senior nuclear medicine physician/radiologist (ET) and a second radiologist (HS). LNs were scored using the DS, and LNs with a DS of 3, 4, or 5 were considered PET-positive regardless of size. The DS 5-point scoring system is an internationally accepted clinical tool based on the comparison between lesion and reference organ uptake of [18F]-FDG. DS 3 is defined as an uptake > mediastinal blood pool but ≤ liver [18, 19]. DS 4 is defined as an uptake moderately increased compared to liver.

Patients with PET-positive regional or common iliac/para-aortic (CI/PA) LNs (*n* = 103) constituted the present study population.

There is currently no consensus or clear international guidelines on how to define PET-positivity in LNs of anal cancer patients. Garda et al. used uptake > background liver (corresponding to DS 4) and Dapper et al. used a combination of different factors to define PET-positive LNs [21, 22]. In Sweden, uptake > mediastinal blood pool (corresponding to DS 3) is commonly used. We therefore decided to use DS 3 as cut-off for our primary analysis and DS 4 as cut-off for our sensitivity analysis.

Primary tumor localization was retrospectively coded into the sub-groups listed in Table 1 based on findings on clinical examination and radiology. The perianal component was defined as any tumor extension outside the anal verge. Rectal component was defined as any tumor extension above the puborectalis muscle.

**Table 1:** Number of patients with PET-positive lymph nodes in different regions; in all patients and in subgroups according to primary tumor location

Regions of PET-positive lymph nodes						
	Inguinal^a^	Perirectal^a^	Internal iliac^a^	External iliac	Common iliac	Para-aortic
All patients (*n* = 103)	75^b^ *of* 103 (73%)	35 (34%)	28 (27%)	33 (32%)	11 (11%)	9 (9%)
Tumor localization						
Anal canal	6 *of* 10 (60%)	3 (30%)	1 (10%)	3 (30%)	3 (30%)	1 (10%)
Anal canal + rectum	11 *of* 32 (34%)	17 (53%)	15 (47%)	13 (41%)	4 (13%)	3 (9%)
Anal canal + perianal	32 *of* 33 (97%)	2 (6%)	1 (3%)	8 (24%)	0 (0%)	1 (3%)
Anal canal + rectum + perianal	26 *of* 28 (93%)	13 (46%)	11 (39%)	9 (32%)	4 (14%)	4 (14%)

^a^ *P* < 0.001 in crosstabs of lymph node positivity in relation to primary tumor localization

^b^ 47 unilateral and 28 bilateral

Detailed information on PET-CT acquisition as well as LN characterization and mapping, including DS, is available in Additional file 1.

### Lymph node regions

Each LN was allocated to one of the following ten nodal regions: inguinal (left/right), external iliac (left/right), internal iliac (left/right), common iliac (left/right), perirectal, and para-aortic. A solitary region was defined as PET-positive LNs limited to only one region and could thus be considered a surrogate for first echelon nodes. Each LN was further allocated to one of the sub-regions listed in Table 2. The definitions and boundaries used for LN regions and sub-regions were mainly based on the definitions used by Paño et al. [23] (Additional file 1).

**Table 2:** Number of sub-regions with PET-positive lymph nodes and number of PET-positive lymph nodes in the sub-regions

	Regions with PET-positive lymph nodes, *n*	Number of PET-positive lymph nodes, *n*
Saphenofemoral (L + R)^a^	97	119
Lower inguinal (L + R)	18	22
Upper inguinal (L + R)	26	41
Lower external iliac, lateral (L + R)	1	1
Lower external iliac, medial (L + R)	30	35
Lower external iliac, middle (L + R)	2	2
Upper external iliac, lateral (L + R)	0	0
Upper external iliac, medial (L + R)	6	6
Upper external iliac, middle (L + R)	4	4
Lower internal iliac (L + R)	27	29
Upper internal iliac (L + R)	10	12
Common iliac, lateral (L + R)	8	9
Common iliac, medial (L + R)	6	6
Common iliac, middle (L + R)	3	5
Perirectal	35	77
Para-aortic, left	9	22
Para-aortic, right	3	6
Para-aortic, aortocaval	6	16

Abbreviations: *L* Left; *R* Right

^a^ For bilateral regions, both left and right side counted

### Mapping of lymph nodes

**Fig. 1:**
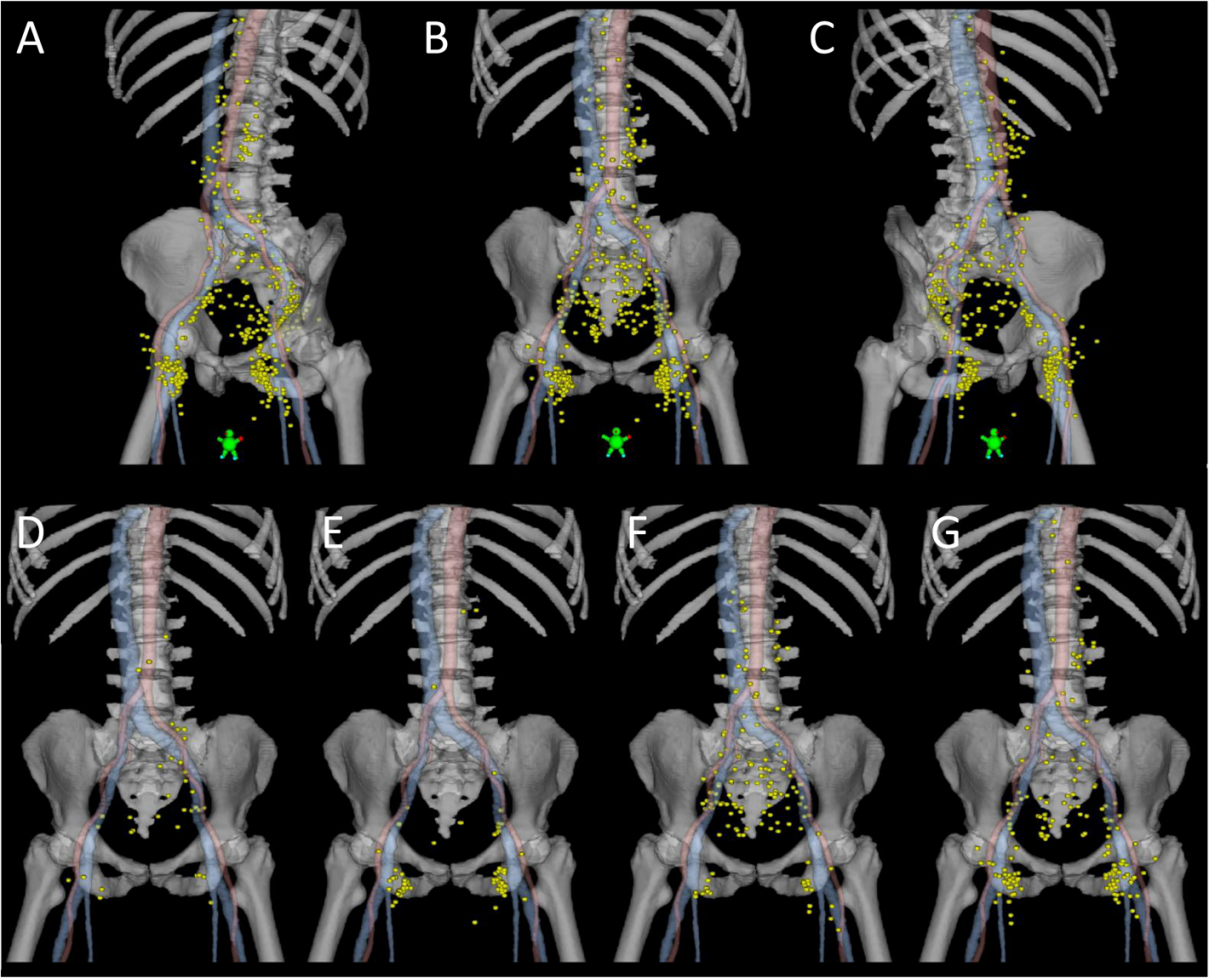
PET-positive lymph nodes (*yellow*) in all patients (**a**-**c**), in patients with anal canal tumors (**d**), anal canal tumors with perianal extension (**e**), anal canal tumors with rectal extension (**f**), and anal canal tumors with both perianal and rectal extension (**g**). Center of lymph node mapped with a 6 mm sphere in a standard anatomy reference CT. *Red*, arteries. *Blue*, veins, including the saphenous vein

**Fig. 2:**
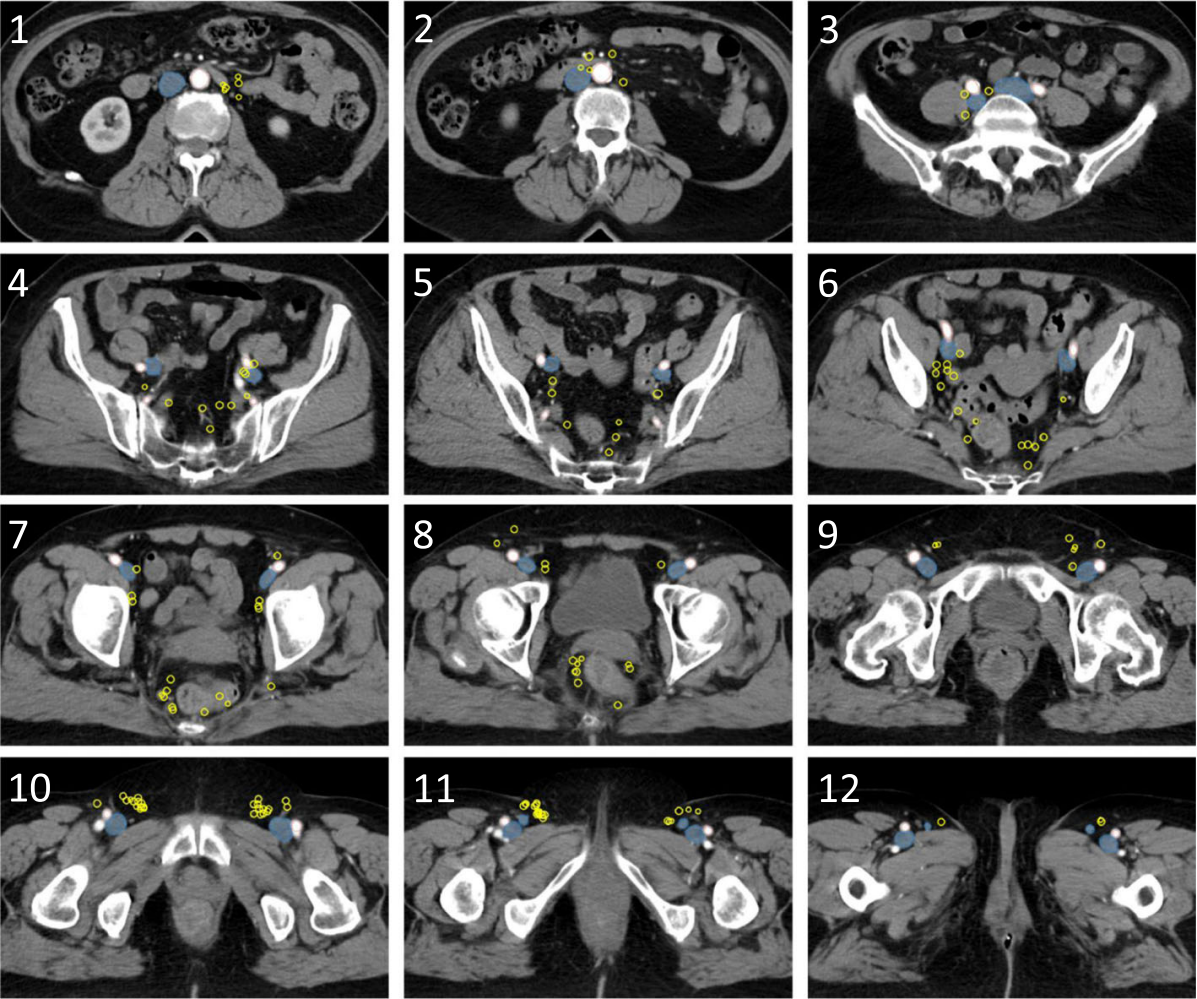
Axial CT slices with PET-positive lymph nodes (*yellow*) in all patients. Center of lymph node mapped with a 6 mm sphere in a standard anatomy reference CT. *Red*, arteries. *Blue*, veins

The center of each PET-positive LN was mapped on a standard anatomy reference CT based on its relation to major arteries and veins (Figs. 1 and 2). The relation to bone, muscles, and skin was also considered. PET-positive LNs on baseline PET-CT were mapped by a radiologist (AF) together with a radiation oncologist (MPN). Specifically, in the cranio-caudal direction, inguinal LNs were mapped based on the level of the saphenous junction defined as the first CT-slice with visible fat separating the saphenous vein and the femoral vein.

### Regions of special interest and skip metastasis

Before the study was initiated, it was determined that the presence or absence of PET-positive LNs in five regions of special interest would be registered and reported. These regions included the ischiorectal fossa; the area located posterolateral to the deep inguinal vessels; along the inferior epigastric vessels superomedial to the inguinal region; the ano-inguinal lymphatic drainage (AILD) [24]; and the area along the internal pudendal vessels lateral to the sacrospinous ligament (Figure in Additional file 3). According to Norwegian contouring guidelines, the cranial border of the elective CTV for T1–2N0 perianal or anal canal tumors without extension into the rectum should be at the bottom of the sacroiliac joint (SIJ) [15, 25]. To evaluate the Norwegian contouring guidelines, PET-positive LNs located above the bottom of the SIJ in patients without PET-positive LNs below the bottom of the SIJ, i.e., a ‘skip metastasis’, were recorded.

### Statistical analysis

Associations between tumor localization and LN positivity in different LN regions, and factors of potential importance for the presence of PET-positive CI/PA LNs (factors selected based on previous results of Nilsson et al. [20]) were visualized using crosstabs. Statistical significance was assessed with a chi-squared test or Fisher’s exact test as appropriate. All significance tests were 2-sided, and *P* values < 0.05 were considered statistically significant. The statistical analysis was conducted in SPSS version 25 (SPSS Inc., Chicago, Illinois, USA).

Sensitivity analyses were performed by changing the cut-off of PET-positivity from DS3 to DS4 and hence restricting the study population to patients with DS4 and DS5 LNs (*n* = 89). This did not change any of the main findings of the study (Additional file 2).

## Results

Baseline PET-CT of 190 anal cancer patients were re-evaluated. The total number of PET-positive LNs defined as DS3–5 was 412 (85 DS3, 173 DS4 and 154 DS5). Depending on DS cut-offs DS3–5, D4–5, and D5; 103/190 (54%), 89/190 (47%) and 44/190 (23%) of patients had PET positive LNs, respectively. Most patients with PET-positive LNs were women (79%), and T1 tumor stage was rare (3%). Patient and tumor characteristics are presented in Supplementary Table 1 (Additional file 1).

### Distribution of PET-positive lymph nodes

Most of the patients with PET-positive LNs had PET-positive LNs in the inguinal region (75 of 103; 73%). Perirectal (34%), internal iliac (27%), and external iliac (32%) PET-positive LNs were also common (Table 1). The pattern of PET-positive LNs was dependent on the localization of the primary tumor (Fig. 1). Comparing anal canal tumors with extension into the rectum and anal canal tumors with a perianal component, the latter more frequently had inguinal, and less often perirectal and internal iliac PET positive LNs (*P* < 0.001) (Table 1).

Table 2 demonstrates the distribution of PET-positive LNs for each anatomical sub-region. Notably, very few PET-positive LNs were found in the lateral (1 of 48) or middle (6 of 48) external iliac sub-region.

### Solitary and non-solitary lymph node patterns

PET-positive LNs in a solitary region were found in 42 patients most commonly in the inguinal region (25 of 42; 60%) followed by perirectal (26%), internal iliac (10%), external iliac (2%), and common iliac (2%) (Table 3). Of 25 cases with inguinal solitary region, 24 (96%) had a positive saphenofemoral node (Table 3, footnote). Patients with anal canal tumors with perianal extension most likely had an inguinal solitary region (16 of 17; 94%) in contrast to patients with anal canal tumors with extension into the rectum where perirectal solitary region was more common (9 of 15; 60%) (Table 3).

**Table 3:** Number of patients with PET-positive lymph nodes in a solitary region; in all patients and in subgroups according to primary tumor localization

Solitary region^a^ of PET-positive lymph nodes						
	Inguinal^b^	Perirectal^b^	Internal iliac	External iliac	Common iliac	Para-aortic
All patients (*n* = 42)	25^c^ *of* 42 (60%)	11 (26%)	4^d^ (10%)	1^e^ (2%)	1^f^ (2%)	0 (0%)
Tumor localization						
Anal canal	4 *of* 5 (80%)	0 (0%)	0 (0%)	0 (0%)	1 (20%)	0 (0%)
Anal canal + rectum	2 *of* 15 (13%)	9 (60%)	3 (20%)	1 (7%)	0 (0%)	0 (0%)
Anal canal + perianal	16 *of* 17 (94%)	1 (6%)	0 (0%)	0 (0%)	0 (0%)	0 (0%)
Anal canal + rectum + perianal	3 *of* 5 (60%)	1 (20%)	1 (20%)	0 (0%)	0 (0%)	0 (0%)

^a^ PET-positive lymph nodes limited to only one of the following regions: left inguinal, right inguinal, perirectal, left internal iliac, right internal iliac, left external iliac, right external iliac, left common iliac, right common iliac, para-aortic

^b^ *P* < 0.01 in crosstabs of lymph node positivity in relation to tumor localization

^c^ 21 saphenofemoral (SF); 2 SF + upper inguinal; 1 SF + lower inguinal; 1 upper inguinal

^d^ 3 lower internal iliac; 1 upper internal iliac

^e^ 1 lower external iliac, medial

^f^ 1 common iliac, lateral. This lymph node is likely a false positive, further explained in the results section

No patient had solitary LNs in the para-aortic region and only one patient had solitary LNs in the common iliac region. Importantly, this single solitary LN in the common iliac region was likely a false positive because it remained stationary on several CT studies prior and after the diagnosis and treatment of anal cancer. The LN was not included in the radiotherapy planning target volume, but the patient remained recurrence-free at the last follow up three years after the end of radiotherapy.

### PET-positive lymph nodes in the common iliac and para-aortic region

Thirteen patients had PET-positive LNs in the CI/PA region (Table 1). Significant associations were found between PET-positive CI/PA LNs and PET-positive LNs in ≥3 other LN regions (*P* = 0.04), in the external iliac region (*P* = 0.01), and in the internal iliac region (*P* = 0.02) (Table 4).

**Table 4:** Common iliac and/or para-aortic (CI/PA) PET-positive lymph nodes in different subgroups

	CI/PA PET-positive lymph nodes^a^		
	No, *n* (%)	Yes, *n* (%)	*P*-value
T stage			0.75^b^
T1–3	61 (86%)	10 (14%)	
T4	29 (91%)	3 (9%)	
Lymph node regions^c^ with PET-positive lymph nodes			**0.04**
< 3 regions	67 (92%)	6 (8%)	
≥ 3 regions	23 (77%)	7 (23%)	
Site of PET-positive lymph nodes			
Inguinal	67 (89%)	8 (11%)	0.33
Internal iliac	21 (75%)	7 (25%)	**0.02**
External iliac	24 (73%)	9 (27%)	**0.004**^b^
Perirectal	29 (83%)	6 (17%)	0.32

^a^ At the time of anal cancer diagnosis; i.e., not recurrence

^b^ Fisher’s exact test; chi square for all other *P*-values

^c^ 7 regions: left inguinal; right inguinal; left internal iliac; right internal iliac; left external iliac; right external iliac; perirectal

### Regions of special interest

No PET-positive LNs were identified in the ischiorectal fossa, posterolateral to deep inguinal vessels, or along the inferior epigastric vessels. One patient had PET-positive LNs (or possibly in transit tumor deposits) located in the AILD. One patient had a PET-positive LN located along the internal pudendal vessels lateral to the sacrospinous ligament (Fig. 2, slice 7).

### Skip metastasis above the bottom of the sacroiliac joint

Forty-three of 103 patients with PET-positive LNs had a primary tumor that did not extend into the rectum. Of these, only one had a PET-positive LN above the bottom of the SIJ without having PET-positive LNs below the SIJ. That patient was the patient with the solitary common iliac LN described in the section above. Thus, the LN was most likely a false positive. Accordingly, no patient in our cohort without primary tumor extension into the rectum had a skip metastasis above the bottom of the SIJ. In fact, also among patients with a primary tumor extension into the rectum it was infrequent; only 1 of 60 had a skip metastasis above the bottom of the SIJ.

## Discussion

We conducted a detailed analysis of patterns of LN spread in anal cancer based on findings on baseline PET-CT. The results could be of importance in future revisions of anal cancer contouring guidelines.

A clear difference in the patterns of PET-positive LNs was noted depending on the location of the primary tumor (Fig. 1). The first echelon nodes (corresponding to solitary region LNs) of lower tumors were primarily inguinal whereas the first echelon nodes of more superiorly located tumors were more often perirectal or internal iliac. These highly expected results support the use of PET-CT for assessing patterns of LN spread in anal cancer, adding validity to the other – more novel – findings of our study.

Our results suggest that the elective CTV might be reduced for some anal cancer patients. According to Norwegian contouring guidelines, the cranial border of the elective CTV for T1–2N0 low tumors (perianal or anal canal tumors without extension into the rectum) should be at the bottom of the SIJ [25]. This recommendation is inconsistent to RTOG and Australasian guidelines—both of these recommend the bifurcation of the common iliac vessels to be the cranial border in all patients [13, 14]. In our cohort, no patient with a low tumor had a skip metastasis above the bottom of the SIJ supporting the less extensive Norwegian guidelines [15]. Furthermore, we did not find a single case of PET-positive LNs in the ischiorectal fossa favoring the UK and RTOG guidelines rather than the Australasian recommendation regarding elective CTV coverage of the entire ischiorectal fossa. Finally, no cases of PET-positive LNs were seen in the inguinal area located posterolateral to the deep vessels. To date, all anal cancer contouring guidelines have included this area in the elective CTV [13–16]. However, our findings are consistent with recent studies by Dapper et al. [22] and Garda et al. [21] and suggest that this area can be omitted from the elective CTV.

Vilarino et al. used USPIO-MRI to identify non-metastatic pelvic LNs in patients with gynecologic malignancies [26]. The mean number of LNs located lateral to the external iliac vessels was 9.5 per patient. Moreover, these LNs were often located > 8 mm from the vessels. Some anal cancer contouring guidelines have therefore recommended a larger margin anterolateral to the external iliac vessels [13]. In our study, only 1 of 412 (0.2%) PET-positive LNs was located in the lateral external iliac sub-region. In fact, most PET-positive external iliac LNs were located posterior to the external iliac vein, particularly below the SIJ (Table 2, Fig. 2). Involvement of the external iliac LNs was relatively common (32% of patients with PET-positive nodes), but only 2% had solitary involvement of the external iliac region. Taken together, we conclude that although LNs are often located lateral to the external iliac vessels, these LNs are very seldom metastatic in anal cancer patients. We also conclude that the external iliac region is not a common first metastatic site in anal cancer.

In a previous study, we investigated the patterns of recurrence in 170 anal cancer patients treated with curative intent—most of whom were included in the present study [20]. In that study, PET-positive LNs in ≥3 LN regions and external iliac metastasis were associated with an increased risk of CI/PA recurrence. In the present study, both of these variables were associated with the presence of PET-positive CI/PA nodes at baseline. Thus, variables that were associated with microscopic tumor spread, i.e., recurrence of nodes that were negative at baseline, were also associated with macroscopic tumor spread at baseline, i.e., PET-positivity. The current results support the conclusion from the previous study; namely that patients with certain patterns of metastatic pelvic LNs might be at an increased risk of harboring tumor cells in the CI/PA region. These regions, or at least parts of these regions, should perhaps be included in the elective CTV for these patients. However, the results need to be replicated in other studies before any recommendations could be made, preferably with prospective study designs.

The results of our study might also have implications for radiologists and nuclear medicine physicians. Deciding whether a LN is pathologic or not is currently a great challenge. The ESGAR guidelines on LN assessment in rectal cancer encourages the radiologist to consider the size and shape of the LN as well as the signal intensity (for MRI studies) [27]. Despite using these assessment tools, there is still a considerable amount of uncertainty regarding many LNs. Deep insight into metastatic patterns in malignant tumors could advise radiologists regarding complex cases. For example, this could facilitate assessment of ambiguous CI/PA nodes not only by the appearance but also by the pattern of pelvic nodes. The absence of PET-positive pelvic LNs decreases the likelihood of the ambiguous CI/PA nodes being metastatic—the presence of multiple PET-positive pelvic LNs increases the probability of CI/PA metastasis. The final decision as to whether a LN should be regarded as pathologic should be taken at a multidisciplinary conference where oncologists, radiologists/nuclear medicine physicians and surgeons work to conclude what is most beneficial for the individual patient.

To the best of our knowledge, this is the largest study to date on patterns of PET-positive LNs in anal cancer. The cohort consists of consecutive patients, and PET-CT was performed regardless of tumor stage. The systematic reassessment of all imaging studies is also a strength. DS was used to give a quantitative and consistent interpretation of the FDG-uptake. The DS is more stable than (for example) the standardized uptake value (SUV) that varies more with different PET-scanners and reconstruction algorithm [18]. DS3 was chosen as a cut-off because FDG-uptake > mediastinal blood is commonly considered to be pathologic [18]. Changing the cut-off from DS3 to DS4 (FDG-uptake > liver) did not impact any of the main findings.

There are also limitations to our study. First, the following presumptions were made: PET-positivity correlates with macrometastasis, and macrometastasis at a later stage of disease correlates with micrometastasis at an earlier stage of the disease. Without pathologic evidence, we cannot be certain that all mapped LNs were true nodal metastases and we point out that our study does not address the question of what the optimal FDG-uptake cut-off should be for LNs in anal cancer. However, the patterns of PET-positive LNs in relation to tumor location concur with a previous study that used other methods for assessing LN metastasis, e.g., lymphatic mapping and sentinel LN biopsy [28]. Second, the center of each LN was mapped as a surrogate for the location of the LN before it was enlarged. Although this assumption possibly is not correct for all LNs, this method has commonly been used by other researchers [21, 22]. Third, the cohort is not large enough to include all potential rare patterns of LN spread in anal cancer.

## Conclusions

Our results suggest some modifications to the elective CTV in anal cancer, for instance: 1) The ischiorectal fossa outside the primary tumor CTV could probably be excluded for most patients, 2) The area posterolateral to the deep inguinal vessels could be omitted, and 3) The cranial border of the elective CTV might be lowered to the bottom of the SIJ for patients with T1–2N0 tumors not extending into the rectum. These changes would lead to slightly reduced volumes of normal tissue being irradiated, which could contribute to a reduction in radiation side-effects.

## Supplementary Information

**Additional file 1: Supplementary Table S1.** with patient and tumor characteristics, and detailed descriptions of PET-CT acquisition, lymph node characterization, anatomic landmarks for lymph node regions, and mapping of lymph nodes.

**Additional file 2. **Sensitivity analyses with study cohort restricted to patients with Deauville score 4/5 lymph nodes on baseline PET-CT (*n* = 89).

**Additional file 3: Supplementary Figure S1.** with regions of special interest.

## Data Availability

The present data is summarized in this paper. The complete dataset can be retrieved from the corresponding author on reasonable request.

## Abbreviations

AILD: Ano-inguinal lymphatic drainage
CI/PA: Common iliac/para-aortic
CT: Computed tomography
DS: Deauville score
IMRT: Intensity-modulated radiotherapy
LN: Lymph node
PET: Positron emission tomography
SIJ: Sacroiliac joint
SUV: Standardized uptake value
