# Supplementary material for: Patterns of pathologic lymph nodes in anal cancer: a PET-CT-based analysis with implications for radiotherapy treatment volumes
Source: BMC Cancer. 2021 Apr 22;21:447. doi: 10.1186/s12885-021-08187-8 (PMC8063376; doi:10.1186/s12885-021-08187-8)
Supplement: Supplementary file 1 — Additional file 1: Supplementary Table S1. with patient and tumor characteristics, and detailed descriptions of PET-CT acquisition, lymph node characterization, anatomic landmarks for lymph node regions, and mapping of lymph nodes. [file 12885_2021_8187_MOESM1_ESM.docx]

**Additional file 1**

| **Supplementary Table S1**. Patient and tumor characteristics. | | | |
| --- | --- | --- | --- |
|  | Entire anal cancer cohort (*n* = 203), *n* (%) | Patients with baseline PET-CT (*n* = 190), *n* (%) | Patients with PET-positive lymph nodes^a^ on baseline PET-CT (*n* = 103), *n* (%) |
| Age at diagnosis (years), Median; range | 64.6; 44.1-92.6 | 64.3; 44.1-92.6 | 64.3; 44.1-90.1 |
| Female | 160 (79%) | 151 (79%) | 81 (79%) |
| HIV | 2 (1%) | 2 (1%) | 2 (2%) |
| Tumor localization |  |  |  |
| Anal canal | 39 (19%) | 33 (17%) | 10 (10%) |
| Anal canal + rectum^b^ | 59 (29%) | 58 (31%) | 32 (31%) |
| Anal canal + perianal^c^ | 68 (33%) | 63 (33%) | 33 (32%) |
| Anal canal + rectum + perianal^d^ | 37 (18%) | 36 (19%) | 28 (27%) |
| T stage^e^ |  |  |  |
| 1. | 20 (10%) | 18 (9%) | 3 (3%) |
| 2. | 93 (46%) | 85 (45%) | 37 (36%) |
| 3. | 45 (22%) | 44 (23%) | 31 (30%) |
| 4. | 45 (22%) | 43 (23%) | 32 (31%) |
| Lymph node metastasis, N+^f^ | 105 (52%) | 99 (52%) | 92 (89%) |
| Distant metastasis, M1^f,g^ | 17 (8%) | 15 (8%) | 14 (14%) |
| Postoperative radiotherapy^h^ | 10 (5%) | 8 (4%) | 1 (1%) |
| ^a^ Deauville score 3-5 |  |  |  |
| ^b^ Tumor extension above puborectalis muscle; also includes patients with tumor in rectum without extension to the anal canal | | | |
| ^c^ Tumor extension outside anal verge; also includes patients with perianal tumor without extension into the anal canal | | | |
| ^d^ Tumor extension both above puborectalis muscle and outside anal verge | | |  |
| ^e^ TNM8 |  |  |  |
| ^f^ As judged by the treating clinicians at the time of diagnosis | |  |  |
| ^g^ M1 includes common iliac and para-aortic lymph node metastasis | | |  |
| ^h^ Following surgical resection without any macroscopic tumor left | | |  |

***PET-CT acquisition***

Three different imaging systems were used for PET-CT-scanning. GE Discovery MI, GE Discovery 690 and Philips Gemini TF. The scans were performed in either normal or frog-legged planning position.

All patients received an intravenous injection of 4 MBq/kg body weight of [18F]-FDG with an accumulation time of 60 min before imaging and after at least 4 h of fasting and a glucose level ≤ 10 mM. Patients were scanned from the central part of the femur to the base of the skull. Acquisition time was 1.5-2 min per bed position (depending on imaging system used). Acquisition was carried out with time-of-flight enabled for all systems. Image reconstructions were performed according to routine clinical practice with reconstruction parameters to obtain the best clinical image per system. For the D690, the ordered subset expectation maximization reconstruction algorithm was used with 3 iterations and 12 subsets, a 5-mm Gaussian post filter, and a 192 × 192 matrix (pixel size 3.6 × 3.6 mm^2^, slice thickness 3.3 mm). For the DMI, the block-sequential regularized expectation maximization reconstruction algorithm was used with a β value of 550 and a 256 × 256 matrix (pixel size 2.7 × 2.7 mm^2^, slice thickness 2.8 mm). For the Gemini TF, the line-of-response row-action maximum likelihood algorithm method was used with a 144 × 144 matrix (pixel size 4.0 × 4.0 mm^2^ and slice thickness 4.0 mm). All PET-CTs were cross-calibrated to the same dose calibrator, and the calibration was validated monthly in a standardized uptake value (SUV) control with phantom.

A diagnostic CT with intravenous and oral contrast or a low-dose CT without contrast was performed for attenuation correction and anatomic correlation of the PET images. In our clinical routine, the PET-scan is combined with a low-dose CT if a previous diagnostic CT was not executed within 4 weeks. The number of CT slices obtained varied (16-128) depending on the current imaging system used.

***Lymph node characterization***

The baseline PET-CT study for all 103 patients with PET-positive lymph nodes (LNs) was analyzed with reference to the location of pathological LNs, metabolic parameters, and hence Deauville score (DS). The DS 5-point scoring system is an internationally accepted clinical tool based on the comparison between lesion and reference organ uptake of [18F]-FDG [1]. The DS is widely used to assess treatment response in lymphoma [2] but has also been used to investigate treatment response in other clinical settings [3].

The standardized uptake value (SUV) is the ratio between the radiotracer concentration in a voxel or group of voxels and the injected activity divided by a normalization factor; in clinical practice, this is usually body weight. The SUVmax is the uptake in the single voxel exhibiting the highest tracer uptake in a region of interest (ROI). SUVmax is easily available, has good inter-reader reproducibility, and is relatively unaffected by partial volume effects. Differences in SUVs caused by differences in image acquisition parameters (scanner, scatter and attenuation correction, reconstruction algorithm) compromise the comparison of SUVs acquired at different centers. This problem is reduced when normalizing the lesion SUV to a reference organ. Therefore, lesion-to-reference organ SUV ratios rather than direct SUV values should be used when comparing results from different scanners [2].

The maximum SUV (SUV_max_) of the liver, mediastinal blood pool and LNs were measured for each patient using the application toolbox in Sectra PACS software (Sectra AB, Linköping, Sweden). LN SUV_max_ was compared to SUV_max_ in the liver and blood pool in order to assign a DS to each LN. The different Deauville scoring levels are defined as follows:

- DS1: no uptake above the background
- DS2: uptake ≤ mediastinal blood pool
- DS3: uptake > mediastinal blood pool but ≤ liver
- DS4: uptake moderately increased compared to liver
- DS5: uptake markedly increased to liver

The quantitative defining criteria for DS5 have been discussed earlier [1, 4], and the 2014 guidelines for response assessment of lymphoma recommends that DS5 be applied to uptake two to three times the uptake in normal liver [5]. In our study, we categorized LNs with an uptake ≥ two times the liver SUVmax as DS5. Our definition of DS4 LNs was an uptake greater than the liver SUVmax. LNs with a DS of 3-5 were considered PET-positive. We did not include LNs with a DS < 3 in our analysis. The cut-off at DS 3 was chosen because this equals an uptake higher than the mediastinal blood pool, which is often considered to be an elevated uptake [2].

***Lymph node regions***

*Inguinal*

The inguinal region was defined as the area below the level of the inguinal ligament. The inguinal ligament was defined as the top of the femoral head where the external iliac artery changes direction and enters the pelvis. The saphenofemoral junction was defined as the first CT-slice with visible fat separating the saphenous vein and the femoral vein. An inguinal node was regarded as a saphenofemoral node if it is located medial to the saphenous junction, within 10 mm above and below the saphenofemoral junction. LNs located below this level were named lower inguinal nodes. LNs above the saphenofemoral level were considered as upper inguinal nodes. In case of doubt, the radiologist decided whether the LN was saphenofemoral or not.

*External iliac*

The external iliac region was defined as the area between the inguinal ligament and the common iliac arterial bifurcation. This region was divided into three sub-regions: medial, lateral, and middle. The medial chain contained LNs located medial and posterior to the external iliac vein. The middle chain comprised LNs located between the external iliac artery and the external iliac vein including LNs that were anteromedial to the artery. The lateral chain included nodes that were located along the lateral aspect of the external iliac artery. This description has been proposed by Paño and Taylor [6, 7]. We also included LNs located anterior/anterolateral to the external iliac artery in the lateral chain. The sub-regions were then further divided into an upper and a lower part where the limit between the two was half the distance of the entire region as specified above. Upper iliac nodes were categorized either external or internal depending on the vessel that was closest to the LN exempt for ‘junctional nodes’ located in the junction between the external and internal vessels; these were categorized as upper *external* nodes [8]. The LNs in between the internal and external vessels were classified as external iliac if they were *anterior* to the ureter and as internal iliac if they were *posterior* to the ureter according to the definition proposed by Lengelé et al. [8]. In cases where the ureter could not be identified, the midpoint of the lateral pelvic wall was used to separate anterior from posterior nodes.

*Internal iliac*

The internal iliac region was defined as the area between the pelvic floor and the common iliac arterial bifurcation [9].This region was further divided into an upper and a lower level where the limit between the two was half the distance of the entire region as specified above.

*Common iliac*

The common iliac region was defined as the area between the common iliac arterial bifurcation and the aortic bifurcation. This region was divided into three sub-regions: medial, lateral, and middle as described by Paño et al. [6].

*Para-aortic*

The para-aortic region was defined as the area between the aortic bifurcation and the renal vein. This region was divided into three sub-regions: aorto-caval, left para-aortic, and right para-caval as described by Takiar et al. [10].

*Perirectal*

Perirectal nodes included LNs in the mesorectum, presacral position, and nodes adjacent to the superior rectal artery. The mesorectum was defined as the area demarcated by the mesorectal fascia and in the cranio-caudal direction by the rectosigmoid junction/the superior rectal artery and anorectal junction, respectively. Presacral nodes were nodes outside the mesorectal fascia close to the sacrum but regarded as neither a node in the rectal superior region nor the internal iliac region.

***Mapping of lymph nodes***

LNs in Deauville category 3-5 were transferred onto a CT scan in the planning software ARIA version 15.6 (Varian Medical Systems, Palo Alto, CA). For CT template, we selected a CT study of a diseased patient from our cohort with ’standard anatomy’. In this template, the exact level (slice) corresponding to the anatomical regions and sub-regions described above were determined aiming for a precise delineation of all nodal regions.

Four identical templates were made and named according to the location of the primary tumor: anal canal, anal canal/rectum, anal canal/perianal, anal canal/perianal/rectum. PET-positive LNs were mapped in either one of the templates depending on primary tumor site.

The center of each LN was mapped using a spherical 6 mm diameter three-dimensional brush. The brush size was the same for all nodes not dependent on the actual LN size.

To transfer the LNs to the template as accurately as possible, the anatomical conditions of each PET-positive LN were considered, i.e., the relation to vessels as well as both bony and soft tissue structures. Perirectal nodes were mapped based on the relation to the anorectal junction (caudally), the rectosigmoid junction and the superior rectal artery (cranially), the rectum, and the sacral bone. For external iliac, common iliac, and para-aortic LNs, the mapping was performed based on the distance to the border of the closest vessel. For internal iliac LNs, the distance to the closest vessel was measured. Bony and muscular anatomy was also considered because the internal iliac branches vary substantially between people and are also hard to visualize on a CT. For inguinal LNs, mapping in the axial plane was based on the distance to the border of the closest vessel. However, for LNs located close to the skin, the distance to the skin was also taken into consideration. In the cranio-caudal direction, inguinal LNs were mapped based on the level of the saphenous junction defined as the first CT-slice with visible fat separating the saphenous vein and the femoral vein. The four templates were fused into one single template creating an overview that is an easily accessible visual presentation of all PET-positive LNs regardless of primary tumor level.

**References**

1. Meignan, M., et al., *Report on the First International Workshop on Interim-PET-Scan in Lymphoma.* Leuk Lymphoma, 2009. **50**(8): p. 1257-60.

2. Barrington, S.F. and R. Kluge, *FDG PET for therapy monitoring in Hodgkin and non-Hodgkin lymphomas.* Eur J Nucl Med Mol Imaging, 2017. **44**(Suppl 1): p. 97-110.

3. Kollberg, P., et al., *[(18)F]Fluorodeoxyglucose-positron emission tomography/computed tomography response evaluation can predict histological response at surgery after induction chemotherapy for oligometastatic bladder cancer.* Scand J Urol, 2017. **51**(4): p. 308-313.

4. Hasenclever, D., et al., *qPET - a quantitative extension of the Deauville scale to assess response in interim FDG-PET scans in lymphoma.* Eur J Nucl Med Mol Imaging, 2014. **41**(7): p. 1301-8.

5. Barrington, S.F., et al., *Role of imaging in the staging and response assessment of lymphoma: consensus of the International Conference on Malignant Lymphomas Imaging Working Group.* J Clin Oncol, 2014. **32**(27): p. 3048-58.

6. Paño, B., et al., *Pathways of lymphatic spread in male urogenital pelvic malignancies.* Radiographics, 2011. **31**(1): p. 135-60.

7. Taylor, A., et al., *Mapping pelvic lymph nodes: guidelines for delineation in intensity-modulated radiotherapy.* Int J Radiat Oncol Biol Phys, 2005. **63**(5): p. 1604-12.

8. Lengelé, B. and P. Scalliet, *Anatomical bases for the radiological delineation of lymph node areas. Part III: Pelvis and lower limbs.* Radiother Oncol, 2009. **92**(1): p. 22-33.

9. Valentini, V., et al., *International consensus guidelines on Clinical Target Volume delineation in rectal cancer.* Radiother Oncol, 2016. **120**(2): p. 195-201.

10. Takiar, V., et al., *Anatomic distribution of fluorodeoxyglucose-avid para-aortic lymph nodes in patients with cervical cancer.* Int J Radiat Oncol Biol Phys, 2013. **85**(4): p. 1045-50.
