# Supplementary material for: Patterns of pathologic lymph nodes in anal cancer: a PET-CT-based analysis with implications for radiotherapy treatment volumes
Source: BMC Cancer. 2021 Apr 22;21:447. doi: 10.1186/s12885-021-08187-8 (PMC8063376; doi:10.1186/s12885-021-08187-8)
Supplement: Supplementary file 2 — Additional file 2. Sensitivity analyses with study cohort restricted to patients with Deauville score 4/5 lymph nodes on baseline PET-CT (n = 89). [file 12885_2021_8187_MOESM2_ESM.pdf]

Sensitivity analyses: study cohort restricted to patients with Deaville score 4/5 lymph nodes on baseline PET-CT (*n* = 89)

Supplementary Table S1. Patient and tumor characteristics.

	Entire anal cancer cohort (*n* = 203), *n* (%)	Patients with baseline PET-CT (*n* = 190), *n* (%)	Patients with Deauville 3/4/5 lymph nodes on baseline PET-CT (*n* = 103), *n* (%)	Patients with Deauville 4/5 lymph nodes on baseline PET-CT (*n* = 89), *n* (%)
Age at diagnosis (years)				
Median; range	64.6; 44.1-92.6	64.3; 44.1-92.6	64.3; 44.1-90.1	63.9 (44.1-90.1)
Female gender	160 *of* 203 (79%)	151 *of* 190 (79%)	81 *of* 103 (79%)	71 *of* 89 (80%)
Tumor localization				
Anal canal	39 (19%)	33 (17%)	10 (10%)	7 (8%)
Anal canal + rectum[a]	59 (29%)	58 (31%)	32 (31%)	31 (35%)
Anal canal + perianal[b]	68 (33%)	63 (33%)	33 (32%)	25 (28%)
Anal canal + rectum + perianal[c]	37 (18%)	36 (19%)	28 (27%)	26 (29%)
T stage[d]				
1	20 (10%)	18 (9%)	3 (3%)	2 (2%)
2	93 (46%)	85 (45%)	37 (36%)	31 (35%)
3	45 (22%)	44 (23%)	31 (30%)	26 (29%)
4	45 (22%)	43 (23%)	32 (31%)	30 (34%)
Lymph node metastasis, N+[e]	105 (52%)	99 (52%)	92 (89%)	83 (93%)
Distant metastasis, M1[e,f]	17 (8%)	15 (8%)	14 (14%)	14 (16%)
Postoperative radiotherapy[g]	10 (5%)	8 (4%)	1 (1%)	0 (0%)

[a] Tumor extension above puborectalis muscle; also includes patients with tumor in rectum without extension to the anal canal

[b] Tumor extension outside anal verge; also includes patients with perianal tumor without extension into the anal canal

[c] Tumor extension both above puborectalis muscle and outside anal verge

[d] TNM8

[e] As judged by the treating clinicians at the time of diagnosis

[f] M1 includes common iliac and para-aortic lymph node metastasis

[g] Following surgical resection without any macroscopic tumor left

Table 1a. Number of patients with PET-positive lymph nodes (Deauville 4/5) in different regions; in all patients and in subgroups according to primary tumor location

	Regions of pathologic lymph nodes					
	Inguinal[a]	Perirectal[a]	Internal iliac[b]	External iliac	Common iliac	Para-aortic
All patients (*n* = 89)	62 *of* 89 (70%)	31 (35%)	25 (28%)	22 (25%)	9 (10%)	8 (9%)
Tumor localization						
Anal canal (*n* = 7)	5 *of* 7 (71%)	2 (29%)	1 (14%)	2 (29%)	1 (14%)	1 (14%)
Anal canal + rectum (*n* = 31)	9 *of* 31 (29%)	17 (55%)	14 (45%)	8 (26%)	4 (13%)	3 (10%)
Anal canal + perianal (*n* = 25)	24 *of* 25 (96%)	1 (4%)	1 (4%)	5 (20%)	0 (0%)	1 (4%)
Anal canal + rectum + perianal (*n* = 26)	24 *of* 26 (92%)	11 (42%)	9 (35%)	7 (27%)	4 (15%)	3 (12%)

[a] $P < 0.001$ in crosstabs of lymph node positivity in relation to tumor localization

[b] $P = 0.002$ in crosstabs of lymph node positivity in relation to tumor localization

Table 1b. Number of patients with PET-positive lymph nodes (Deauville 4/5) in a solitary region; in all patients and in subgroups according to primary tumor localization

	Solitary region[a] of pathologic lymph nodes					
	Inguinal[b]	Perirectal[b]	Internal iliac	External iliac	Common iliac	Para-aortic
All patients (*n* = 38)	21 *of* 38 (55%)	11 (29%)	3 (8%)	3 (8%)	0 (0%)	0 (0%)
Tumor localization						
Anal canal (*n* = 4)	3 *of* 4 (75%)	0 (0%)	0 (0%)	1 (25%)	0 (0%)	0 (0%)
Anal canal + rectum (*n* = 16)	2 *of* 16 (13%)	9 (56%)	3 (19%)	2 (13%)	0 (0%)	0 (0%)
Anal canal + perianal (*n* = 13)	12 *of* 13 (92%)	1 (8%)	0 (0%)	0 (0%)	0 (0%)	0 (0%)
Anal canal + rectum + perianal (*n* = 5)	4 of 5 (80%)	11 (29%)	0 (0%)	0 (0%)	0 (0%)	0 (0%)

[a] Pathologic lymph nodes limited to only one of the following regions: left inguinal, right inguinal, perirectal, left internal iliac, right internal iliac, left external iliac, right external iliac, left common iliac, right common iliac, para-aortic

[b] $P \leq 0.02$ in crosstabs of lymph node positivity in relation to tumor localization

Table 2. Number of sub-regions with PET-positive lymph nodes (Deauville 4/5) and number of pathologic lymph nodes in the sub-regions.

	Regions with pathologic lymph nodes, *n*	Number of pathologic lymph nodes, *n*
Saphenofemoral (L+R)[a]	78	92
Lower inguinal (L+R)	16	19
Upper inguinal (L+R)	22	30
Lower external iliac, lateral (L+R)	1	1
Lower external iliac, medial (L+R)	20	23
Lower external iliac, middle (L+R)	2	2
Upper external iliac, lateral (L+R)	0	0
Upper external iliac, medial (L+R)	6	6
Upper external iliac, middle (L+R)	2	2
Lower internal iliac (L+R)	24	26
Upper interla iliac (L+R)	8	9
Common iliac, lateral (L+R)	6	7
Common iliac, medial (L+R)	6	6
Common iliac, middle (L+R)	2	3
Perirectal	31	62
Para-aortic, left	7	17
Para-aortic, right	3	6
Para-aortic, aortocaval	6	16

Abbreviations: L, left; R, right

[a] For bilateral regions, both left and right side counted

Table 3. Common iliac and/or para-aortic (CI/PA) PET-positive lymph nodes (Deauville 4/5) in different subgroups

	CI/PA metastasis[a]		
	No, *n* (%)	Yes, *n* (%)	*P*-value
T stage			0.74[b]
T1-3	51 (86%)	8 (14%)	
T4	27 (90%)	3 (10%)	
Lymph node regions[c] with metastasis			0.01
< 3 regions	62 (93%)	5 (7%)	
≥ 3 regions	16 (73%)	6 (27%)	
Site of lymph node metastasis			
Inguinal	55 (89%)	7 (11%)	0.73[b]
Internal iliac	19 (76%)	6 (24%)	0.04
External iliac	17 (81%)	5 (23%)	0.09
Perirectal	25 (81%)	6 (19%)	0.14

[a] At the time of anal cancer diagnosis; i.e., not recurrence

[b] Fisher´s exact test; chi square for all other *P*-values

[c] 7 regions: left inguinal; right inguinal; left internal iliac; right internal iliac; left external iliac; right external iliac; perirectal
