# Supplementary material for: Patterns of pathologic lymph nodes in anal cancer: a PET-CT-based analysis with implications for radiotherapy treatment volumes
Source: BMC Cancer. 2021 Apr 22;21:447. doi: 10.1186/s12885-021-08187-8 (PMC8063376; doi:10.1186/s12885-021-08187-8)
Supplement: Supplementary file 3 — Additional file 3: Supplementary Figure S1. with regions of special interest. [file 12885_2021_8187_MOESM3_ESM.pptx]

## Slide 1
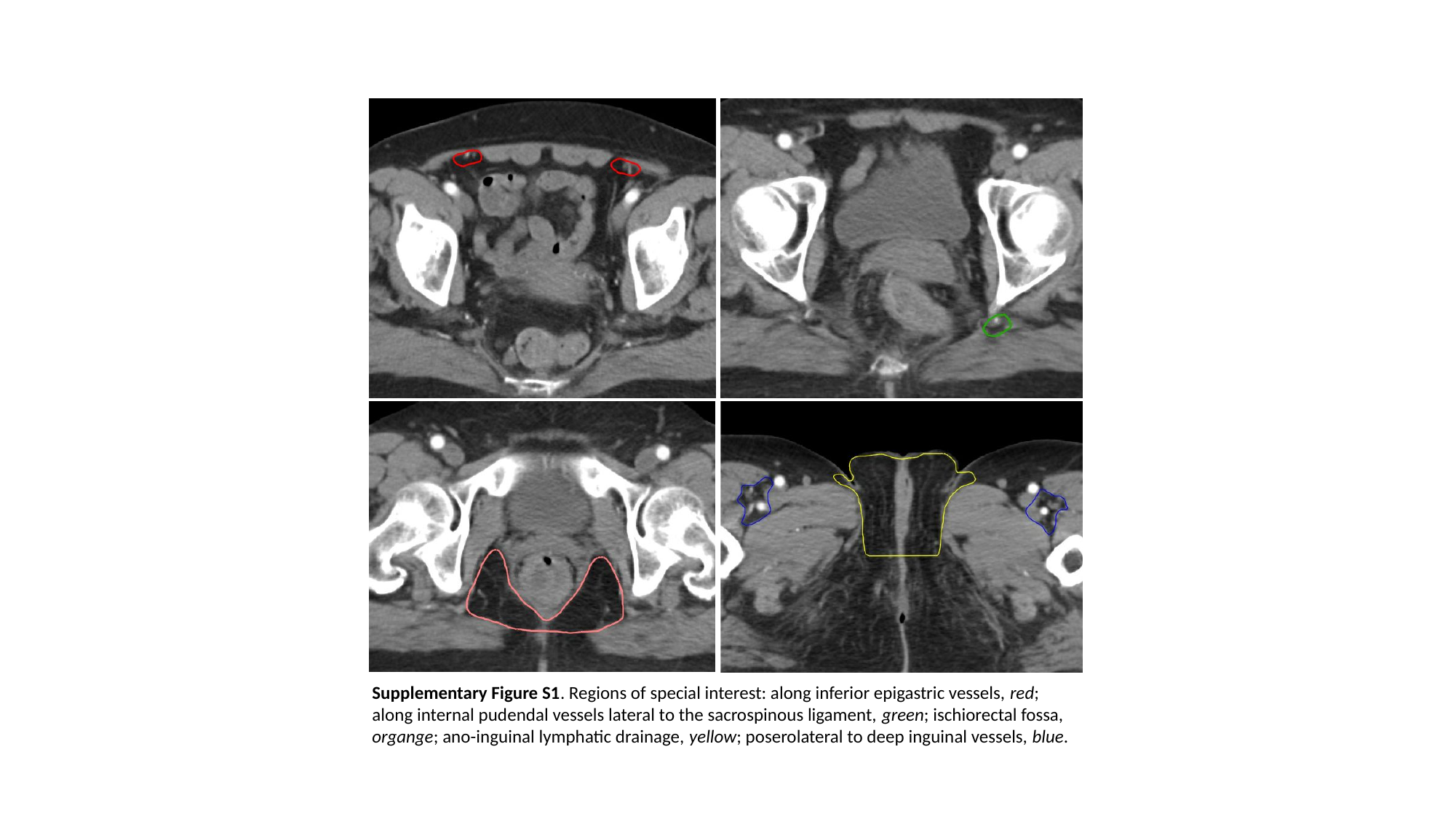

Supplementary Figure S1. Regions of special interest: along inferior epigastric vessels, red; along internal pudendal vessels lateral to the sacrospinous ligament, green; ischiorectal fossa, organge; ano-inguinal lymphatic drainage, yellow; poserolateral to deep inguinal vessels, blue.
